# Atypical T1 Hyperintense Neurocysticercosis Masquerading As Cystic Brain Metastases

**DOI:** 10.1200/JGO.2016.006536

**Published:** 2016-08-31

**Authors:** Abhishek Mahajan, Mehul Patel, Nilesh Sable, Meenakshi H. Thakur

**Affiliations:** All authors: Tata Memorial Centre, Mumbai, Maharashtra, India

## INTRODUCTION

Neurocysticercosis (NCC) is a clinical condition characterized by involvement of the CNS by an encysted larval form of the parasite *Taenia solium*. It is a common cause of seizures and neurologic morbidity in developing countries.^1,2^ Brain metastasis is a common complication of cancer, and cystic brain lesions often pose a diagnostic challenge in this clinical scenario.^3^ The imaging appearance of NCC on magnetic resonance imaging (MRI) depends on the stage of the disease. To our knowledge, cystic lesions in NCC appearing hyperintense on T1-weighted, T2-weighted, and fluid-attenuated inversion recovery (FLAIR) images have not been described in the literature. We report one such a case, with multiple intracranial cystic lesions showing hyperintense signals on T1-weighted images.

## CASE REPORT

**Fig 1:**
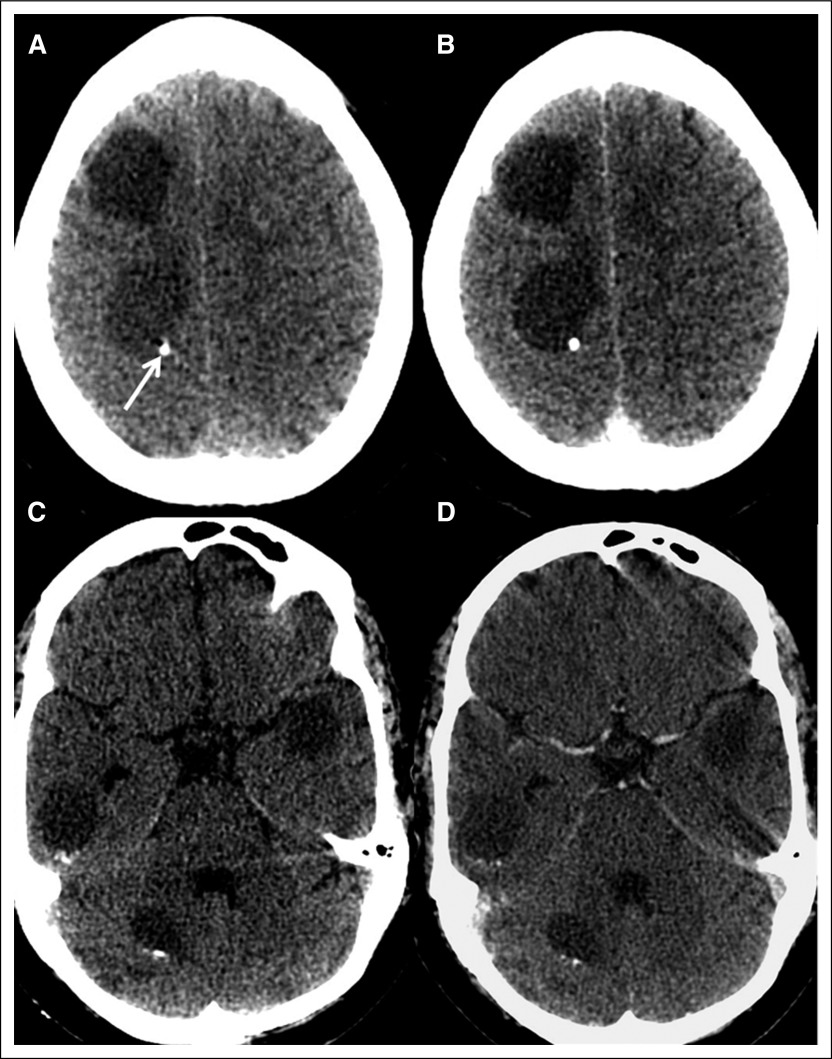
(A and C) Plain and (B and D) postcontrast computed tomography images showing multiple nonenhancing hypodense lesions in the bilateral cerebral and cerebellar hemispheres. These lesions were cystic (nonenhancing hypodense areas), and a few of them showed eccentric calcification (hyperdense foci; arrow).

**Fig 2:**
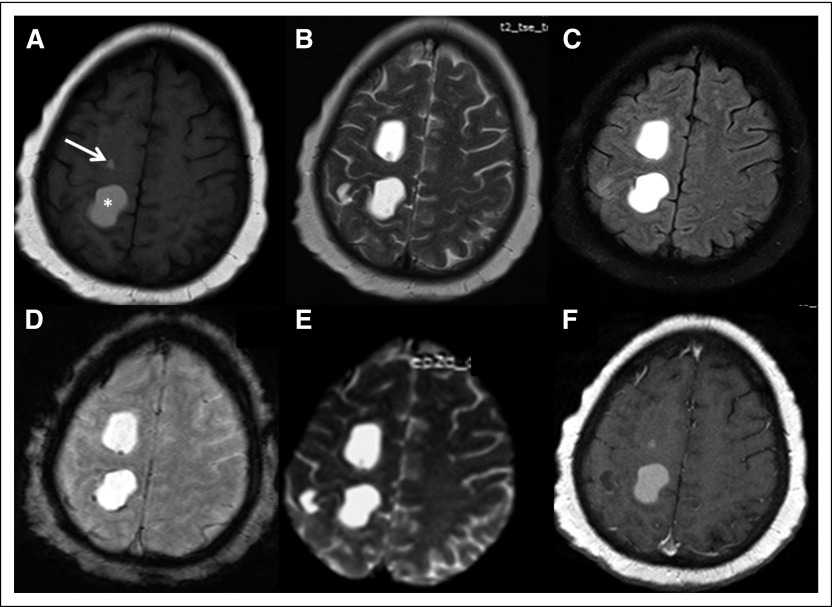
(A) T1-weighted, (B) T2-weighted, (C) fluid-attenuated inversion recovery, (D) susceptibility-weighted, (E) *m*-ADC (mean apparent diffusion coefficient) map, and (F) postcontrast T1-weighted magnetic resonance images showing multiple cystic lesions in the right cerebral hemisphere. The cyst followed fluid signal on magnetic resonance imaging (hypointense to isointense relative to CSF on T1-weighted images, hyperintense on T2-weighted images, and suppressed on fluid-attenuated inversion recovery images). A few of the cystic lesions were completely hyperintense on T1-weighted images (asterisk), and a few showed eccentric hyperintense focus on T1-weighted imaging (arrow) that on susceptibility-weighted images revealed eccentric focal blooming corresponding to the calcification seen on CT imaging; no hemorrhage was seen within. None of the lesions showed diffusion restriction, perilesional edema, or enhancing solid component within.

**Fig 3:**
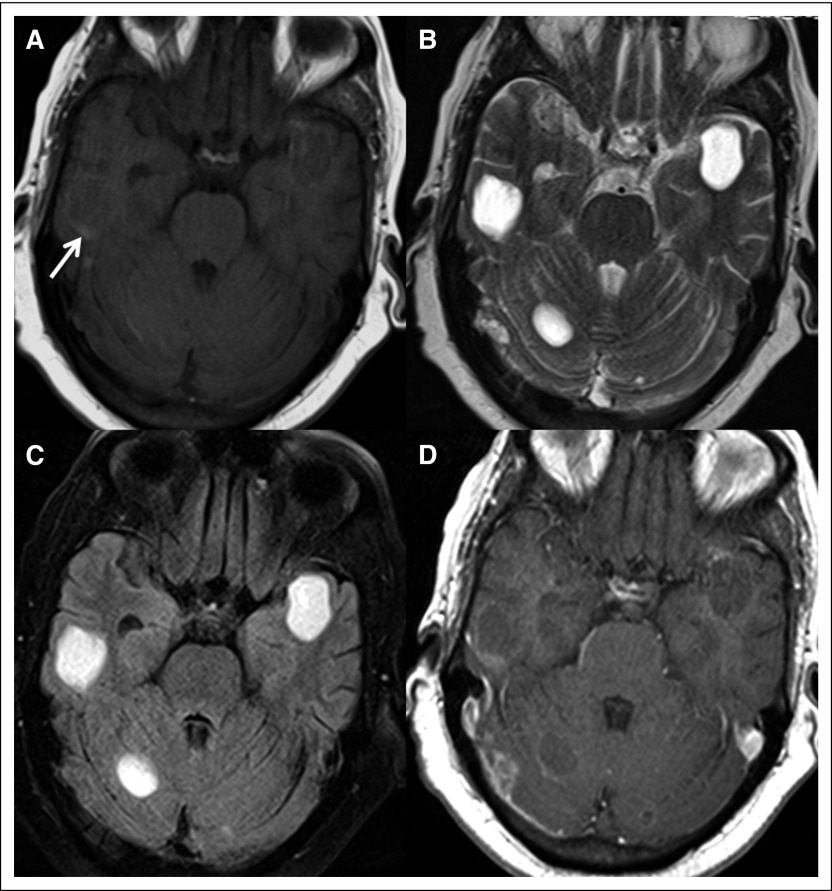
(A) T1-weighted, (B) T2-weighted, (C) fluid-attenuated inversion recovery, and (D) postcontrast T1-weighted magnetic resonance images showing multiple cystic lesions in the cerebral and cerebellar hemispheres. The cyst followed fluid signal on magnetic resonance imaging (hypointense to isointense relative to CSF on T1-weighted, hyperintense on T2-weighted, and suppressed on fluid-attenuated inversion recovery images). A few lesions showed eccentric hyperintense focus on T1-weighted imaging (arrow) that on susceptibility-weighted images revealed eccentric focal blooming corresponding to calcification seen on CT imaging; no hemorrhage was seen within. None of the lesions showed diffusion restriction or perilesional edema. Thin ring enhancement was seen in a few of the lesions.

A 63-year-old woman with breast cancer and lung, bone, nodal, and skin metastases presented with a history of headache and dizziness. She was referred for contrast-enhanced computed tomography (CT) scanning to rule out brain metastases. CT imaging (Fig 1) revealed multiple hypodense lesions in the bilateral cerebral and cerebellar hemispheres, with attenuation values of approximately 17 to 18 Hounsfield units. These lesions showed no significant perilesional edema or postcontrast enhancement. Most of these cystic lesions, however, showed eccentric calcifications. On the basis of the imaging findings, a provisional diagnosis of NCC with cystic brain metastasis was made. Magnetic resonance imaging (MRI) of the brain was recommended to evaluate the nature of the parenchymal lesions seen on CT scans. MRI (Figs 2 and 3) revealed multiple cystic lesions in the bilateral cerebral and cerebellar hemispheres. Most of these lesions appeared hypointense on T1-weighted images and hyperintense on T2-weighted and FLAIR images. A few of these were hyperintense on T1-weighted, T2-weighted, and FLAIR sequences. None of them showed diffusion restriction, perilesional edema, or a significant enhancing cystic solid component. A few of the lesions showed subtle, thin, peripheral rim enhancement. Susceptibility-weighted imaging (SWI) revealed eccentric focal blooming in most of these cystic lesions, corresponding to the calcification seen on the CT scan. No evidence of any intracranial metastases was noted. The features suggested NCC, but an unusual finding was the hyperintense signal in the cystic component of a few lesions seen on T1-weighted images (asterisk in Fig 2) and focal eccentric T1-weighted hyperintense foci (arrow in Fig 2A) in a few lesions. A final diagnosis of NCC was made, and the patient was treated with albendazole therapy.

## DISCUSSION

Presence of multiple cystic lesions in the brain carry a wide range of differential diagnosis (Table 1), and knowledge of imaging patterns is important to accurately diagnose these lesions and differentiate them from their close mimics.^2,3^ The diagnosis depends on imaging findings (CT and MRI), CSF studies, and clinical correlation. The imaging diagnosis in such lesions depends on the imaging pattern and clinical-epidemiologic background. NCC is a common systemic parasitic infection affecting the nervous system, muscles, and other soft tissues. Clinical presentation ranges from trivial symptoms, such as headache, vomiting, and fever, to seizures, focal neurologic deficit, and stiff neck. Because there is no specific clinical sign to suggest a diagnosis of NCC, imaging by CT scan or MRI is the mainstay of the diagnostic work-up.^1^ Brain metastasis is a common complication of cancer, and differentiating these lesions from NCC may be challenging in the presence of an atypical imaging pattern.^1,2^

**Table 1:**
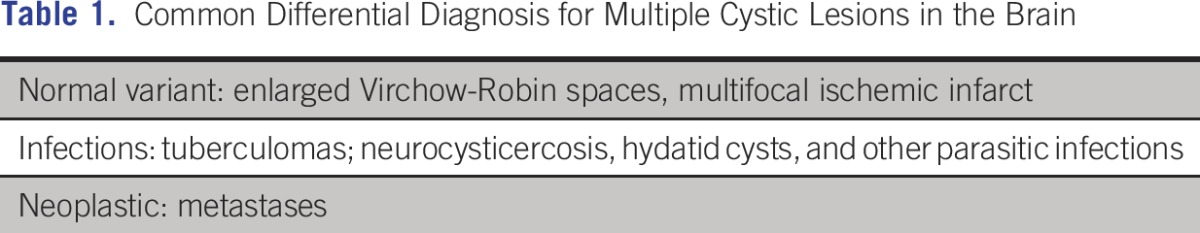
Common Differential Diagnosis for Multiple Cystic Lesions in the Brain

The imaging appearance of NCC on MRI depends on the stage of the disease. The four recognized stages of NCC are vesicular, colloidal vesicular, granular nodular, and nodular calcified.^2,4^ The vesicular stage on CT/MRI appears as a well-defined cyst with a thin perceptible wall and no perilesional edema or postcontrast enhancement. The cyst follows fluid signal on both the CT scan (isoattenuating to CSF) and MRI (hypointense to isointense relative to CSF on T1-weighted images, hyperintense on T2-weighted images, and suppressed on FLAIR images).^4,5^ An eccentrically located discrete scolex within the cyst characterizes the stage. The parasite larva is viable but escapes the host immune response in this stage. Thereafter, the larva degenerates, with features of hyaline degeneration. The cyst shrinks in size, and fluid becomes turbid, with proteinaceous content inciting the inflammatory response from the host. This progresses into the colloidal vesicular stage, characterized by cyst fluid contents that appear slightly hyperattenuating on CT compared with CSF and follows a fluid intensity pattern on MRI. This stage is characterized by the appearance of ring enhancement and perilesional edema. The granular nodular stage shows retraction and mineralization of the lesion. On imaging, partial regression of the edema and postcontrast peripheral enhancement are shown. The nodular calcified stage is characterized by complete mineralization of the lesion. The cyst becomes calcified, with no edema or postcontrast enhancement. The lesion is typically hypointense on T1-weighted and T2-weighted sequences and calcified on CT scan.^2,4,5^ Because there are no specific clinical signs to suggest the diagnosis of NCC, imaging by CT scan or MRI is the mainstay of diagnostic work-up. Incidental findings of multiple calcifications in brain parenchyma were the main imaging findings in the initial studies.^4^ Comparisons between the diagnostic ability of CT and MRI are well established. MRI has been shown to be superior to CT imaging in the detection of parenchymal as well as ventricular NCC.^2,4^ CT scanning, however, better delineates the calcifications in the later stages.^2,4^ In atypical cases, laboratory testing of the serum using enzyme-linked immunoelectrotransfer blot and the CSF using enzyme-linked immunosorbent assay or enzyme-linked immunoelectrotransfer blot-2 aids the diagnosis.^4,5^

In our case, the fluid component of a few lesions showed hyperintense signal on T1-weighted imaging, and a few lesions showed eccentric hyperintense signal on T1-weighted imaging. The cause of T1-weighted hyperintensity in NCC has not been well described in the literature. Small eccentric T1-weighted hyperintense areas may occur as a result of the paramagnetic effect of the soft calcification in the scolex of the larva, which on gradient recalled echo/SWI appears as a hypointense signal and corresponds to hyperdense calcified areas on CT.^6^ The hyperintensity of the cystic fluid component of the lesion can be secondary to degeneration of the encysted larval form. The cyst fluid becomes turbid with an increased amount of proteinaceous material, which explains the hyperintense signal on T1-weighted sequence and can be considered an early sign of degeneration of cysticercus larva and its transformation into the colloidal vesicular stage. In our case, the final diagnosis of NCC was made, and the patient was treated with albendazole therapy.

Our case highlights the importance of always considering neuroinfection in the differential diagnosis of patients with known extracranial malignancy in endemic regions. In conclusion, in developing countries where enzyme-linked immunosorbent assay and immunoblot essays for NCC are not routinely available, knowledge of this atypical imaging pattern has great clinical implications for a timely diagnosis and appropriate management.
